# Hypothalamic arcuate nucleus glucokinase regulates insulin secretion and glucose homeostasis

**DOI:** 10.1111/dom.13359

**Published:** 2018-06-12

**Authors:** Yue Ma, Risheka Ratnasabapathy, Chioma Izzi‐Engbeaya, Marie‐Sophie Nguyen‐Tu, Errol Richardson, Sufyan Hussain, Ivan De Backer, Christopher Holton, Mariana Norton, Gaelle Carrat, Blanche Schwappach, Guy A. Rutter, Waljit S. Dhillo, James Gardiner

**Affiliations:** ^1^ Section of Endocrinology and Investigative Medicine, Division of Diabetes Endocrinology and Metabolism, Imperial College London London UK; ^2^ Section of Cell Biology and Functional Genomics, Division of Diabetes Endocrinology and Metabolism, Imperial College London London UK; ^3^ Department of Molecular Biology, Centre for Biochemistry and Molecular Cell Biology Heart Research Centre Göttingen, University Medicine Göttingen Göttingen Germany

**Keywords:** glucokinase activator, glycaemic control, neuropharmacology, pharmacogenetics

## Abstract

**Aims:**

To investigate the role of arcuate glucokinase (GK) in the regulation of glucose homeostasis.

**Materials and methods:**

A recombinant adeno‐associated virus expressing either GK or an antisense GK construct was used to alter GK activity specifically in the hypothalamic arcuate nucleus (arc). GK activity in this nucleus was also increased by stereotactic injection of the GK activator, compound A. The effect of altered arc GK activity on glucose homeostasis was subsequently investigated using glucose and insulin tolerance tests.

**Results.:**

Increased GK activity specifically within the arc increased insulin secretion and improved glucose tolerance in rats during oral glucose tolerance tests. Decreased GK activity in this nucleus reduced insulin secretion and increased glucose levels during the same tests. Insulin sensitivity was not affected in either case. The effect of arc GK was maintained in a model of type 2 diabetes.

**Conclusions:**

These results demonstrate a role for arc GK in systemic glucose homeostasis.

## 1 INTRODUCTION

Glucokinase (GK) is a member of the hexokinase family^1^ with important roles in glucose sensing and disposal. There are two isoforms of GK, a hepatic form, expressed exclusively in the liver, and a neuroendocrine form expressed in pancreatic β cells and the central nervous system (CNS).^2^ These two isoforms have identical kinetic properties and differ only in the promoter utilized.^3^

In the CNS, GK is expressed in neurons, astrocytes and tanycytes. In neurons it is co‐expressed with GLUT‐2 and ATP‐sensitive potassium channels (K_ATP_)^4^, ^5^ and is part of the glucose‐sensing mechanism,^6^, ^7^ acting in a manner analogous to its role in pancreatic β cells.^2^, ^8^ It is thought that the role of GK‐expressing astrocytes and tanycytes is to confer glucose sensitivity to neurons, which are not themselves glucose‐sensitive.^9^, ^10^

Glucokinase is widely expressed in the CNS. Within the hypothalamus it is present in several nuclei, including the ventromedial nucleus (VMN) and arcuate nucleus (arc).^11^, ^12^, ^13^ GK in the VMN regulates the counter‐regulatory response to hypoglycaemia.^14^, ^15^, ^16^

The arc also has an important role in the regulation of glucose homeostasis. There are extensive neural connections between the arc and pancreas.^17^, ^18^ Importantly, GK activity in the arc appears to be regulated by glucose levels because GK levels decline in the arc of streptozotocin‐treated rats whilst levels in other nuclei are unaffected.^19^

Both glucose excitatory and glucose inhibitory neurons have been demonstrated in the arc and shown to be insulin‐responsive.^16^ These neurons may underlie the response to insulin in the arc, as acute administration of insulin into the arc of rodents reduces hepatic gluconeogenesis and glycogenolysis.^20^ Whilst only a fraction of peripheral insulin crosses the blood–brain barrier, loss of insulin receptors in the brain leads to loss of suppression of hepatic glucose production.^20^, ^21^ This effect is thought to be through Agouti‐related protein (AgRP)‐expressing neurons leading to reduced hepatic gluconeogenesis.^22^

While both the arc and GK are important in regulating glucose homeostasis, the role of GK within the arc in glucose homeostasis has not previously been studied in isolation from other hypothalamic regions. Addressing this question in the present study, we show that arc GK has a role in glucose‐stimulated insulin secretion but does not regulate insulin sensitivity.

## 2 METHODS

### 2.1 Animals

Adult male Wistar rats (230–280 g, Charles River UK Ltd) and 6‐week‐old male Zucker Diabetic Fatty (ZDF) rats (Fa/Fa; Charles River France Ltd) were individually housed and maintained in a controlled environment (temperature 21 °C–23 °C, 12‐hours light–dark cycle, lights on at 07:00 hours). They had ad libitum access to standard chow (RM1 diet; Special Diet Services UK Ltd, Witham, UK) and water. All animal procedures were approved under the British Home Office Animals (Scientific Procedures) Act 1986 (Project Licence no. 70/7229).

### 2.2 rAAV production

rAAV (serotype‐2) encoding either full‐length sense GK (rAAV‐GK), antisense GK (rAAV‐ASGK) or green fluorescent protein (GFP; rAAV‐GFP) were produced as previously described.^23^

### 2.3 Intra‐arcuate rAAV microinjection

A total of 0.5 μL of rAAV‐ASGK (titre: 3.42 × 10^12^ genome particles/mL), rAAV‐GK (titre: 2.96 × 10^12^ genome particles/mL) or rAAV‐GFP (titre: 5.04 × 10^12^ genome particles/mL) was bilaterally injected into the arc of male Wistar rats using coordinates determined from Paxinos and Watson,^24^ as previously described.^23^ No rats were excluded from the analysis.

### 2.4 Intra‐arc administration of pharmacological agents

A permanent stainless steel cannula was inserted unilaterally into the arc as previously described.^23^ Subsequently, the rats were fasted overnight and the following morning injected with one of the following in a volume of 0.5 μL; saline, 0.5 nmol compound A [CpdA; a GK activator, 2‐Amino‐5‐(4‐methyl‐4H‐[1,2,4]‐triazole‐3‐yl‐sulfanyl)‐N‐(4‐methyl‐thiazole‐2‐yl)benzamide] CAS 603108–44‐7 (Merck‐Millipore, Beeston, UK), 1 nmol diazoxide (a K_ATP_ activator) or 2 nmol of glibenclamide (a K_ATP_ blocker). These doses were identical to those used previously.^23^ Thirty minutes after the injection the rats underwent an oral glucose tolerance test (OGTT), as described below. The experiment was a cross‐over design, with each rat receiving each injection; the injections were performed in a random order at least 3 days apart.

At the end of the study, cannula placement was confirmed with Indian ink.^23^ All cannulae were confirmed as correctly placed and no rats were excluded from the analysis.

### 2.5 Oral glucose tolerance tests

Based on our previous findings, OGTTs were performed 3 to 4 weeks after injection of rAAV before significant changes in body weight and food intake occurred to prevent their confounding effects on the measurement of blood glucose.

The rats were acclimatized to drinking glucose solution, were fasted overnight and a 24‐gauge cannula was inserted into the tail vein. The baseline blood sample was collected 1 hour after insertion of the cannula. Then, 2.5 g/kg of glucose (20% w/v) was administered orally to each rat. Following glucose consumption, blood was collected at 15, 30, 60 and 120 minutes. Plasma was separated by centrifugation at 13 000 ×*g* for 5 minutes at 4 °C and was stored at −80 °C.

### 2.6 Insulin tolerance tests

Rats underwent an insulin tolerance test (ITT) 5 weeks after surgery. A 24‐gauge/19‐mm cannula was inserted into the tail vein. The baseline blood sample was collected at 0 minutes. Two units/kg of insulin was injected intraperitoneally. Blood was taken at 15, 30, 60 and 120 minutes. Plasma was separated by centrifugation at 13 000 ×*g* for 5 minutes at 4 °C and was stored at −80 °C.

### 2.7 Collection of tissue samples

Unless otherwise stated in the methods, the rats from all studies were killed in the early light phase. Pancreas, ileum and brain were collected from all rats after the completion of a study.

### 2.8 GK activity assay in isolated hypothalamic nuclei

The brains from the rAAV study groups were used to measure changes in GK activity in hypothalamic nuclei. The arc, VMN and paraventricular nucleus (PVN) were collected by punch biopsy, and GK activity was measured as previously described.^23^

### 2.9 Measurement of glucose, insulin and active glucagon‐like peptide‐1 in plasma samples

Plasma glucose levels were measured using a glucose oxidase assay (Randox, Crumlin, UK) and plasma insulin levels were analysed using a Ultra‐sensitive rat Insulin ELISA Kit from Crystal Chem (Zaandam, Netherlands).

Plasma active glucagon‐like peptide‐1 GLP‐1 level was measured using a glucagon‐like peptide‐1 (Active) ELISA from Millipore (Billerica, Massachusetts) according to the manufacturer's instructions.

### 2.10 Statistical analysis

All data are shown as mean ± SEM. Analysis was by either one‐way or two‐way analysis of variance (ANOVA; as appropriate) with a post hoc Holm–Sidak test or *t* test (GraphPad Prism 8.0). Significance was set at *P* < .05 for all analyses.

## 3 RESULTS

### 3.1 Chronic increases in GK activity in the arc improve glucose tolerance in rats

rAAV‐GK was injected into the arc of male Wistar rats (iARC‐GK rats). Controls were injected with rAAV‐expressing GFP (iARC‐GFP rats).

**Figure 1:**
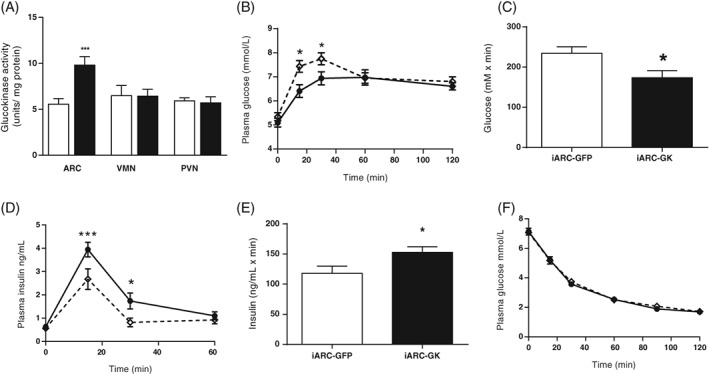
Effect of genetically increased arcuate glucokinase (GK) activity on glucose homeostasis. Groups of adult male Wistar rats were injected with either rAAV‐green fluorescent protein (GFP; iARC‐GFP) or rAAV‐GK (iARC‐GK) bilaterally into the arcuate nucleus. They then underwent an oral glucose tolerance test (OGTT) and an insulin tolerance test (ITT). A, GK activity in arcuate nucleus (ARC), ventromedial nucleus (VMN) and paraventricular nucleus (PVN) of male Wistar rats after intra‐arcuate injection of either rAAV‐GFP (iARC‐GFP, open bars) or rAAV‐GK (iARC‐GK, filled bars). B, Plasma glucose during an OGTT in iARC‐GFP (open diamonds) and iARC‐GK (filled circles) rats. C, Incremental area under the curve analysis of plasma glucose during an OGTT in iARC‐GFP (open bars) and iARC‐GK (filled bars) rats. D, Plasma insulin levels during an OGTT in iARC‐GFP (open diamonds) and iARC‐GK (filled circles) rats, E, Area under the curve analysis of plasma insulin during an OGTT in iARC‐GFP (open bars) and iARC‐GK (filled bars) rats. F, Plasma glucose during an ITT in iARC‐GFP (open diamonds) and iARC‐GK (filled circles) rats. Data are represented as mean ± SEM, *n* = 10. *P < .05, ***P < .001. Data for A, were analysed by analysis of variance (ANOVA) and post hoc Holm–Sidak. Data for B, D and F were analysed by two‐way ANOVA and post hoc Holm–Sidak. Data for C and E were analysed by *t*‐test

The GK activity was increased ~2‐fold specifically in the arc of iARC‐GK rats compared with iARC‐GFP (Figure 1A). GK activity in the VMN and PVN was unaffected (Figure 1A). In support of the specificity of injection site, expression of GFP after rAAV injection was limited to the arc (Figure S1A in File S1).

Food intake and body weight were measured from the day after injection of rAAV for 26 days. There was a trend towards increased body weight (*P* = .08) and food intake (*P* = .15) in iARC‐GK rats compared with iARC‐GFP rats (Figure S1B,C in File S1); however, glucose tolerance tests were performed before significant differences in body weight occurred to prevent its confounding effect on these variables.

During an OGTT iARC‐GK demonstrated 14% lower glucose excursion than control iARC‐GFP rats 15 minutes after ingestion of glucose, 11% lower at 30 minutes and the incremental area under the curve (iAUC) was reduced (Figure 1B,C). Insulin levels were higher in the iARC‐GK group than in the iARC‐GFP group during the OGTT, with insulin levels ~1.5 times higher in the iARC‐GK at 15 minutes and almost 2‐fold higher at 30 minutes, and the iAUC was increased compared with controls (Figure 1D,E). Insulin sensitivity during an ITT (Figure 1F) was unchanged, as were fasting plasma glucose and insulin levels (Figure S1D,E in File S1). Active GLP‐1 levels were the same in iARC‐GK and iARC‐GFP rats during the OGTT (Figure S1F in File S1).

These results suggest that chronically increased GK activity in the arc improves glucose tolerance through increased glucose‐stimulated insulin secretion (GSIS).

Dual immunohistochemistry for GFP and the neuron‐specific protein PGP9.5 showed localization of GFP to neurons. Dual immunohistochemistry for GFP and the astrocyte‐specific protein GFAP did not detect any co‐localization (Figure S2 in File S1).

Western blot analysis was performed on arc samples from rats injected with rAAV expressing either GFP (iARC‐GFP), GK (iARC‐GK) or antisense GK (iARC‐ASGK). There was no significant difference among the groups for expression of either GLUT2 and Kir 6.2 (Figure S3A–C in File S1).

Hypothalamic expression of AgRP was not affected by increased arc GK activity (Figure S3D in File S1).

### 3.2 Enhanced GSIS in rats with chronically increased GK activity in the arc is not attributable to changes in islet cell mass, islet cell insulin synthesis or glucagon

The iARC‐GK rats exhibited equivalent pancreatic weight and β‐cell mass (Figure S4A,B in File S1) compared with controls. Likewise there was no difference in pancreatic insulin expression or content (Figure S4C,D in File S1). Pancreatic proglucagon mRNA levels and content also remained unchanged in iARC‐GK rats compared with iARC‐GFP rats (Figure S4E,F in File S1).

### 3.3 Acute pharmacological activation of arcuate GK activity and blockade of K_ATP_ improves glucose tolerance and activating K_ATP_ worsens glucose tolerance in rats

**Figure 2:**
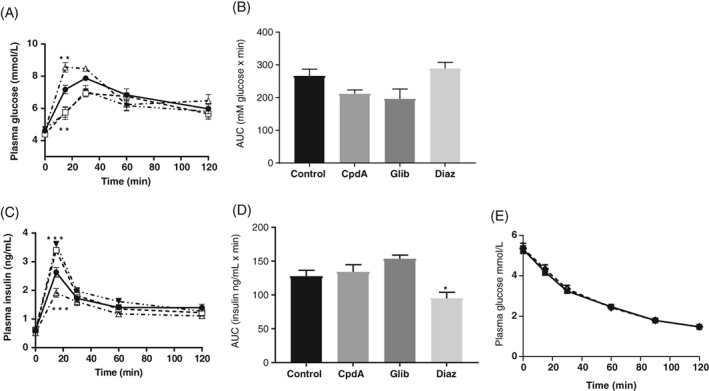
Effect of pharmacologically increased arcuate glucokinase (GK) activity and manipulating K_ATP_ activity on glucose homeostasis in Wistar rats. Adult male Wistar rats were injected into the arcuate nucleus with either vehicle (control) or 0.5 nmol of the GK activator compound A, 2 nmol glibenclamide or 1 nmol diazoxide. A, Plasma glucose during an oral glucose tolerance test (OGTT) in control (filled circle), compound A‐injected (CpdA; open squares), glibenclamide‐injected (Glib; filled inverted triangles) and diazoxide‐injected (Diaz; open triangles) rats. B, Incremental area under the curve (iAUC) analysis of plasma glucose during an OGTT in control, CpdA, Glib or Diaz rats. C, Plasma insulin levels during an OGTT in control (filled circle), CpdA (open square), Glib (filled inverted triangles) and Diaz (open triangles) rats. D, iAUC analysis of plasma insulin during an OGTT in control, CpdA, Glib or Diaz rats. E, Plasma glucose during an insulin tolerance test in iARC‐vehicle‐ (open diamonds) and iARC‐CpdA‐ (filled squares) injected rats. Data are represented as mean ± SEM, *n* = 10. *P < .05. **P < 0.01, ***P < .001, where data points overlay significance refers to both compared to control. Data for A, C and E were analysed by two‐way analysis of variance (ANOVA) and post hoc Holm–Sidak. Data for B and D were analysed by one‐way ANOVA and post hoc Holm–Sidak

Having observed that overexpression of GK in the arc improves glucose tolerance, we aimed to examine the effect of acute pharmacological GK activation and the effect of altering K_ATP_ activity. We therefore measured glucose levels during the OGTT after administration of CpdA (a GK activator), glibenclamide (K_ATP_ blocker) or diazoxide (K_ATP_ activator). CpdA significantly reduced glucose levels at 15 minutes (7.17 ± 0.27 mmol/L iARC‐vehicle vs 5.76 ± 0.31 mmol/L iARC‐CpdA; *P* < .01 [Figure 2A]), but it did not affect the iAUC (Figure 2B). CpdA increased insulin levels at 15 minutes (2.63 ± 0.17 ng/mL iARC‐vehicle vs 3.40 ± 0.10 ng/mL iARC‐CpdA; *P* < .001 [Figure 2C]), but did not affect iAUC (Figure 2D). Glibenclamide significantly reduced glucose levels at 15 minutes (7.17 ± 0.27 mmol/L iARC‐vehicle vs 5.70 ± 0.40 mmol/L iARC‐glibenclamide; *P* < .01 [Figure 2A]), but did not affect iAUC (Figure 2B). Glibenclamide significantly increased insulin secretion at 15 minutes (2.63 ± 0.17 ng/mL iARC‐vehicle vs 3.60 ± 0.11 ng/mL iARC‐glibenclamide; *P* < .001 [Figure 2C]), but did not affect iAUC (Figure 2D). Diazoxide significantly increased glucose levels at 15 minutes (7.17 ± 0.27 mmol/L iARC‐vehicle vs 8.55 ± 0.31 mmol/L iARC‐diazoxide; *P* < .01 [Figure 2A]), but did not alter iAUC (Figure 2B). Diazoxide significantly reduced insulin secretion at 15 minutes (2.63 ± 0.17 ng/mL iARC‐vehicle vs 1.91 ± 0.15 ng/mL iARC‐diazoxide; *P* < .001 [Figure 2C]) and resulted in a reduced iAUC (Figure 2D). Injection of CpdA into the arc did not alter insulin sensitivity during an ITT (Figure 2E).

### 3.4 Chronic decreases in GK activity within the arc impair glucose tolerance in rats

Together, these data show that increased arcuate GK activity improves glucose tolerance by enhancing GSIS. We therefore hypothesized that decreased arcuate GK would impair glucose tolerance through impaired GSIS.

The effect of decreasing arcuate GK activity on glucose homeostasis in rats was studied by stereotactic injection of rAAV encoding antisense GK into the arc (iARC‐ASGK) whilst rAAV‐GFP was injected as a control (iARC‐GFP). The antisense GK construct has previously been shown to specifically reduce GK activity in vivo, with minimal target effects.^23^, ^25^

**Figure 3:**
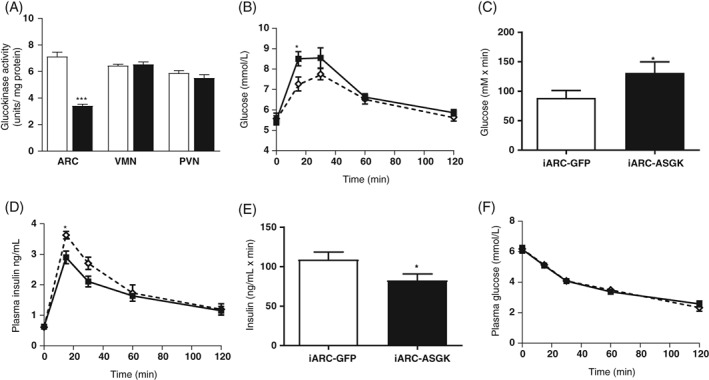
Effect of genetically decreased arcuate glucokinase (GK) activity on glucose homeostasis. Groups of adult male Wistar rats were injected with either rAAV‐green fluorescent protein (GFP; iARC‐GFP) or rAAV‐antisense GK (ASGK; iARC‐ASGK) bilaterally into the arcuate nucleus. They then underwent an oral glucose tolerance test (OGTT) and an insulin tolerance test (ITT). A, GK activity in the arcuate nucleus (ARC), ventromedial and paraventricular nucleus of male Wistar rats following intra‐arcuate injection of either rAAV‐GFP (iARC‐GFP, open bars) or rAAV‐ASGK (iARC‐ASGK, filled bars). B, Plasma glucose during an OGTT in iARC‐GFP (open diamonds) and iARC‐ASGK (filled squares) rats. C, Incremental area under the curve analysis of plasma glucose during an OGTT in iARC‐GFP (open bars) and iARC‐ASGK (filled bars) rats. D, Plasma insulin levels during an OGTT in iARC‐GFP (open diamonds) and iARC‐ASGK (filled squares) rats. E, Area under the curve analysis of plasma insulin during an OGTT in iARC‐GFP (open bars) and iARC‐ASGK (filled bars) rats. F, Plasma glucose during an ITT in iARC‐GFP (open diamonds) and iARC‐ASGK (filled squares) rats. Data are represented as mean ± SEM, *n* = 10. *P < .05, **P < .01, ***P < .001. Data for A were analysed by analysis of variance (ANOVA) and post hoc Holm–Sidak. Data for B, D and F were analysed by two‐way ANOVA and post hoc Holm–Sidak. Data for C and E were analysed by *t* test

The GK activity was decreased ~2‐fold specifically in the arc of iARC‐ASGK rats compared with iARC‐GFP rats (Figure 3A). GK activity in the VMN and PVN was unaffected (Figure 3A).

Glucose levels were 17% higher in iARC‐ASGK than iARC‐GFP rats 15 minutes after glucose ingestion, and iAUC was increased in iARC‐ASGK rats (Figure 3B,C). Insulin levels were 20% lower in the iARC‐ASGK group than in controls at 15 minutes and iAUC was decreased (Figures 3D,E).

We also observed a trend towards a decrease in food intake and body weight in the iARC‐ASGK rats compared with iARC‐GFP, although this did not reach significance during the course of the experiment (Figure S5A,B in File S1).

Insulin sensitivity was unchanged in iARC‐ASGK rats during an ITT (Figure 3F). Fasting glucose (Figure S5C in File S1) and insulin (Figure S5D in File S1) levels were similarly unaffected. Active GLP‐1 levels were not significantly different between iARC‐ASGK and iARC‐GFP rats (Figure S5E in File S1). These results show that GSIS was decreased in iARC‐ASGK rats compared with controls.

### 3.5 Pharmacological activation of arc GK activity improves glucose tolerance in ZDF Fa/Fa rats and the effects of K_ATP_ channel modulators are maintained in them

Having found these effects in normal rats, we examined whether they were maintained in a model of type 2 diabetes, the ZDF Fa/Fa rat. We investigated the effect of arc administration of a GK activator and pharmacological agents which alter activity of the K_ATP_ channel.

**Figure 4:**
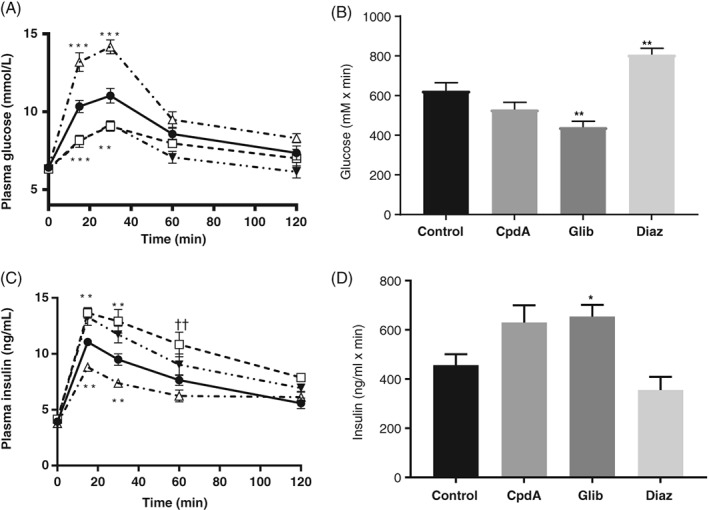
Effect of pharmacologically increased arcuate glucokinase (GK) activity and manipulating K_ATP_ activity on glucose homeostasis in Zucker Diabetic Fatty (ZDF) Fa/Fa rats. Adult male ZDF rats were injected into the arcuate nucleus with either vehicle (control) or 0.5 nmol of the GK activator compound A, 2 nmol glibenclamide or 1 nmol diazoxide. A, Plasma glucose during an oral glucose tolerance test (OGTT) in control (filled circle), compound A‐injected (CpdA; open square), glibenclamide‐injected (Glib; filled inverted triangles) and diazoxide‐injected (Diaz; open triangles) rats. B, Incremental area under the curve (iAUC) analysis of plasma glucose during an OGTT in control, CpdA, Glib or Diaz rats. C, Plasma insulin levels during an OGTT in control (filled circle), CpdA (open square), Glib (filled inverted triangles) and Diaz rats (open triangles). D, iAUC analysis of plasma insulin during an OGTT in control, CpdA, Glib or Diaz rats. Data are represented as mean ± SEM, *n* = 10. *P < .05, **P < .01, ***P < .001, ††*P* < .001 Glib vs control rats, where data point overlay significance refers to both compared with controls. Data for A and C were analysed by two‐way analysis of variance (ANOVA) and post hoc Holm‐Sidak. Data for B and D were analysed by one‐way ANOVA and post hoc Holm–Sidak

In Fa/Fa rats, CpdA lowered glucose levels significantly at 15 minutes (10.34 ± 0.39 mmol/L iARC‐vehicle vs 8.16 ± 0.25 mmol/L iARC‐CpdA; *P* < .001) and 30 minutes (11.03 ± 0.47 mmol/L iARC‐vehicle vs 9.06 ± 0.33 mmol/L iARC‐CpdA; *P* < .01 [Figure 4A]), but it did not affect iAUC (Figure 4B). CpdA increased insulin levels at 15 minutes (11.05 ± 0.28 mmol/L iARC‐vehicle vs 13.65 ± 0.48 mmol/L iARC‐CpdA; *P* < .001), 30 minutes (9.48 ± 0.53 mmol/L iARC‐vehicle vs 12.91 ± 1.06 mmol/L iARC‐CpdA; *P* < .001) and 60 minutes (7.64 ± 0.48 mmol/L iARC‐vehicle vs 10.85 ± 1.10 mmol/L iARC‐CpdA; *P* < .001 [Figure 4C]), but did not affect iAUC (Figure 4D). Similarly glibenclamide significantly decreased glucose levels at 15 minutes (10.34 ± 0.39 mmol/L iARC‐vehicle vs 8.09 ± 0.40 mmol/L iARC‐glibenclamide; *P* < .001) and at 30 minutes (11.03 ± 0.47 mmol/L iARC‐vehicle vs 9.18 ± 0.21 mmol/L iARC‐glibenclamide; *P* < .01 [Figure 4A]) and resulted in a significant decrease in iAUC (Figure 4B). Glibenclamide significantly increased insulin secretion at 15 minutes (11.05 ± 0.28 mmol/L iARC‐vehicle vs 13.29 ± 0.75 mmol/L iARC‐glibenclamide; *P* < .01) and at 30 minutes (9.48 ± 0.53 mmol/L iARC‐vehicle vs 11.73 ± 0.76 mmol/L iARC‐glibenclamide; *P* < .01 [Figure 4C]) and increased the iAUC (Figure 4D).

By contrast, diazoxide significantly raised glucose levels at 15 minutes (10.34 ± 0.39 mmol/L iARC‐vehicle vs 13.19 ± 0.61 mmol/L iARC‐diazoxide; *P* < .001) and at 30 minutes (11.03 ± 0.47 mmol/L iARC‐vehicle vs 14.17 ± 0.45 mmol/L iARC‐diazoxide; *P* < 0.001 [Figure 4A]), and increased the iAUC (Figure 4B). Diazoxide significantly reduced insulin secretion at 15 minutes (11.05 ± 0.28 mmol/L iARC‐vehicle vs 8.81 ± 0.28 mmol/L iARC‐diazoxide; *P* < .01) and at 30 minutes (9.48 ± 0.53 mmol/L iARC‐vehicle vs 7.38 ±0.31 mmol/L iARC‐diazoxide; *P* < .05 [Figure 4C]), but did not affect the iAUC (Figure 4D). Hence, this obese diabetic model has improved glucose homeostasis following arc GK activation and arc sulphonylurea administration, whereas glucose tolerance is worsened by arc administration of K_ATP_ channel activators.

## 4 DISCUSSION

Glucokinase is widely distributed throughout the brain and expressed in both neurons and glia.^5^, ^9^, ^10^, ^11^, ^12^, ^26^ The features of the signalling mechanism in neurons have been characterized in some detail, and the mechanism mirrors that in the pancreatic β cells.^6^, ^7^ A role for VMN GK in the counter‐regulatory response is well documented.^14^, ^27^, ^28^ We recently identified a role for GK within the arc in the regulation of glucose intake.^23^ Using the same model of altering GK activity specifically in the arc using rAAV injections, we investigated its role in the regulation of glucose homeostasis.

Using rAAV we specifically increased and decreased GK activity within the arc and investigated the effect of this on glucose homeostasis. We have previously demonstrated that arc GK regulates body weight and food intake.^23^ We saw similar magnitudes of changes in the present cohort of rats; therefore, we undertook the glucose tolerance tests between 3 and 4 weeks after rAAV injection before these changes had become significant. Altered arc GK activity did not alter fasting plasma glucose or fasting plasma insulin levels; however, differences were apparent during an OGTT. Rats with increased arc GK displayed improved glucose tolerance and increased insulin levels, whilst those with decreased arc GK had worse glucose tolerance and decreased insulin levels. This suggests that the arc GK has a role in regulating GSIS. Fasting glucose and insulin levels were unaffected, which may be attributable to differences in glucose concentrations between the hypothalamus and the circulation. Whilst hypothalamic glucose concentrations reflect plasma levels, they are lower,^29^, ^30^ and glucose entry to the hypothalamus is regulated by nutritional status and insulin.^30^, ^31^ It is therefore possible that fasting levels of glucose within the arc are insufficient to activate GK. Once glucose levels begin to rise after intake of glucose, arc glucose levels rise, GK is activated and acts to reduce the levels of glucose. Our data suggest the main mechanism by which arcuate GK is regulating glucose levels is by changing GSIS; however, sensing of glucose within the hypothalamus is known to regulate hepatic glucose metabolism. It is therefore possible that hepatic glucose metabolism is in part responsible for the effects we observe.^32^, ^33^, ^34^ Changes in insulin sensitivity seem unlikely to be involved as none were detected during the ITT; however, it is possible that minor changes in insulin sensitivity occurred which were not detected under these conditions but might be revealed using alternative approaches. We found no changes in active GLP‐1 levels during the OGTT, suggesting that it is not involved in the altered insulin release.

It is likely that the effect of hypothalamic GK activation on insulin secretion we observed is mediated by the neural connections between the pancreas and GK‐expressing neurons in the arc, as elegantly demonstrated by Stanley et al.^18^ and Rosario et al.^17^. Our data are in accord with those of Osundiji et al.^35^ who reported that infusion of glucose into the third ventricle increased GSIS whilst infusion of a non‐selective GK inhibitor decreased it, although they did not explore which hypothalamic nuclei was involved. Our findings are also in accordance with those of Tarussio et al.^36^ who found that neuron‐selective inactivation of the high Michaelis constant (K_M_) glucose transporter, Glut2/*Slc2a2*, led to impaired glucose tolerance and insulin secretion via lowered parasympathetic nerve activity. The sympathetic nervous system is also important, however, in the hypothalamic regulation of glucose homeostasis.^37^, ^38^, ^39^ It is therefore possible that the sympathetic nervous system is important in the responses we see. Future studies will be necessary to differentiate between these two possibilities.

The findings of our studies are, however, in contrast to those of others.^17^, ^18^ Rosario et al., using adenovirus, showed that increased arc hexokinase 1 (HK1) resulted in decreased glucose sensitivity with decreased insulin release.^17^ There are several possible explanations for this apparent discrepancy. The first possibility is that it reflects differences in the kinetic properties between GK and HK1. HK1 has a low K_M_ for glucose (~0.1 mmoL/L) and is fully active at all physiological glucose concentrations.^40^ It is therefore unlikely that flux through HK1 will be affected by changes in blood (or hypothalamic) glucose concentrations. By contrast, GK has a K_M_ for glucose of ~10 mmol/L, and would thus be expected to respond efficiently over the physiologically relevant range of glucose concentrations.^39^, ^40^ Additionally HK1 is predominantly localized to mitochondria,^13^, ^15^ whilst GK is localized to the cytoplasm in neurons.^4^ These differences in sub‐cellular localization are likely to result in a different metabolic fate for glucose, with alternative signalling pathways activated in response, thus resulting in different physiological responses.^15^ Finally, adenovirus transfects tanycytes and astrocytes as well as neurons.^41^, ^42^ While rAAV type 2, as used in the present study, has a specific tropism for neurons in the brain, this difference is also likely to have functional consequences.

Stanley et al.^18^ used the novel approach of bidirectional electromagnetic stimulation to activate or inhibit GK‐containing neurons in the ventromedial hypothalamus, targeted by selective activation of Cre‐recombinase to determine the utility of this approach for regulating neuronal activity. They demonstrated that activation of these neurons increased plasma glucose and reduced insulin levels, whilst inhibition lowered glucose levels and increased insulin levels. Unlike in the present study, these effects were a combination of activating all forms of GK‐expressing cells, whether they were glucose‐inhibited or glucose‐stimulated, within both the arc and VMN and were independent of the prevailing glucose levels.

Not all arc neurons express GK. To investigate whether activation of endogenous arc GK had the same effect, we injected CpdA, a pharmacological activator of GK, into the arc. As with the virally mediated increases in GK activity, pharmacological activation of the enzyme improved glucose sensitivity during OGTTs. This was associated with increased GSIS but no change in insulin sensitivity. Hence, the effects observed following virally mediated GK over‐expression appear to reflect the physiological function of arc GK in the regulation of glucose homeostasis, rather than being a consequence of introducing GK into cells in which it is not usually expressed.

The evidence we provide that GK in the arc has a physiological role in the regulation of glucose homeostasis is further strengthened by the effect of decreasing GK activity in this brain region. Using a previously validated^23^, ^25^ antisense construct, we reduced GK activity specifically in the arc. This resulted in impaired glucose tolerance and was accompanied by decreased insulin release, effects opposite to those observed with increased GK activity. These results further strengthen the suggestion that GK within the arc may have a physiological role to increase glucose‐stimulated insulin release. These findings, together with our previous data^23^ suggesting that arc GK regulates glucose intake, raise the possibility that GK within the arc coordinates a physiological response to glucose in the diet.

Glucokinase is expressed in both neuropeptide Y (NPY)/AgRP and POMC/CART neurons, although not all. We have previously shown that arcuate GK may be mediating its effects via NPY.^23^ This is consistent with findings in mice with targeted deletion of Kir 6.2, which had increased NPY expression but unaltered POMC expression.^43^ It is therefore possible that arc GK is mediating its effects on glucose via NPY; however, it is also possible that expression of GK in POMC neurons is critical because inhibiting glucose‐sensing POMC neurons by expressing K_ATP_ channels unresponsive to ATP worsens glucose tolerance.^44^ Another possibility is that both types of neuron are important in the observed regulation of glucose homeostasis.

Recent work suggests that the phenotypes of arc nuclei neurons are more complex than previously believed.^45^ The exact identity of the cells that express GK is unclear. Identifying the sub‐types of neurons that express GK and understanding the effects of this co‐localization would be an interesting extension for future work.

In addition to studying rats with normal glucose homeostasis, we also studied ZDF Fa/Fa rats, a model of type 2 diabetes. These demonstrated the same effects and indeed the response observed in the Fa/Fa rats appeared to be of a greater magnitude than in normal rats. This suggests that targeting arc GK would be a useful strategy for the treatment of diabetes.

It follows from our findings that agents which enhance GK activity in the arc are likely to have beneficial effects on glucose metabolism in the context of obesity and type 2 diabetes. The potential benefits of GK activators have been explored before, and several have entered clinical trials since 2008. This class of compounds has shown good efficacy in terms of insulinotrophic, anti‐hyperglycaemic effects and reductions in HbA1c, but none have progressed beyond phase II, mainly because of failure of the therapeutic effect to be maintained.^46^ It is possible that targeting GK activators to the arc could alleviate these problems. It is also possible that agents could be developed that have improved CNS penetrance and, as the arc is in a region with a more porous blood–brain barrier, the agent would have selective access to the arc as compared with other hypothalamic nuclei. Alternatively, it may be possible to use ligand‐directed therapy to target GK activators specifically to the arc. Thus preferential activation of GK in the hypothalamus might ultimately provide a means to reduce the known contra‐indications of using GK activators in these conditions, including elevated hepatic triglyceride production and declining efficacy.^46^

## Supporting information


**Appendix S1**. Immunohistochemistry; Western blot analysis and Quantitative RT‐PCR.
**Figure S1**. Effect of chronically increased glucokinase activity in the arcuate nucleus.
**Figure S2**. Immunohistochemistry localisation of GFP following rAAV injection into the arcuate nucleus.
**Figure S3**. Effect of genetically increased glucokinase activity in the arcuate nucleus on expression of other proteins.
**Figure S4**. Effect of genetically increased glucokinase activity in the arcuate nucleus on islet function.
**Figure S5**. Effect of chronically decreased glucokinase activity in the arcuate nucleus.
