# Supplementary material for: Hypothalamic arcuate nucleus glucokinase regulates insulin secretion and glucose homeostasis
Source: Diabetes Obes Metab. 2018 Jun 12;20(9):2246–54. doi: 10.1111/dom.13359 (PMC6099255; doi:10.1111/dom.13359)
Supplement: Supplementary file 1 — Appendix S1. Immunohistochemistry; Western blot analysis and Quantitative RT‐PCR. Figure S1. Effect of chronically increased glucokinase activity in the arcuate nucleus. Figure S2. Immunohistochemistry localisation of GFP following rAAV injection into the arcuate nucleus. Figure S3. Effect of genetically increased glucokinase activity in the arcuate nucleus on expression of other proteins. Figure S4. Effect of genetically increased glucokinase activity in the arcuate nucleus on islet function. Figure S5. Effect of chronically decreased glucokinase activity in the arcuate nucleus. [file DOM-20-2246-s001.pdf]

Supplementary Appendix

Supplementary methods

Immunohistochemistry

Four weeks after injection of rAAV, the rats were killed with pentobarbitone. Brains were fixed by transcardial perfusion with phosphate-buffered saline (PBS) then 4% phosphate-buffered formaldehyde. Brains were removed, equilibrated in 20% sucrose prior to snap freezing in liquid nitrogen and stored at –80°C until use. Twenty micrometer coronal sections were cut on a freezing sled microtome (Shandon, Runcorn, UK) and mounted on poly-lysine slides. A standard dual immunohistochemistry protocol was performed using the following antibodies mouse anti-GFP antibody (1:500, ab38689) with rabbit anti-PGP9.5 antibody (1:200, ab27053) or mouse anti-GFP antibody with rabbit anti-GFAP antibody (1:500, ab33922). The secondary antibodies donkey anti-rabbit IgG Alexa Fluor® 647 (1:200, ab150075) and goat anti-mouse IgG Alexa Fluor® 488 (1:500, ab150113) diluted in TBS. Cover slips were a fixed using flouroshield mounting medium with DAPI (ab104139). Slides were examined with a Zeiss Axiovert 100 deconvoluting microscope, using an EC Plan-Neofluar 40x/0.75 objective. Images were taken using XX prepared using Image J [1] and no adjustments were made to the images.

Western Blot analysis

The arcuate nucleus of male Wistar rats injected with rAAV were collected by punch biopsy and were lysed in RIPA buffer containing protease inhibitor cocktail tablets (Roche) and centrifuged at 15000g for 15 min to pellet cell debris. Protein concentration was measured using a Pierce BCA protein assay kit (Thermo Fisher Scientific) according to manufacturer's instructions. Twenty-eight micrograms of protein per sample were separated by SDS-PAGE under reducing conditions and transferred onto a polyvinylidene fluoride membrane (0.45 µm, Amersham). The membrane was blocked with 5% milk in PBS-T0.1 (Phosphate Buffer Saline, Tween 0.1%) for 1h and incubated overnight at 4˚C with the primary antibodies anti-GLUT2 (Rabbit, dilution 1/2000) generous gifts from Bernard Thorens, Lausanne or anti-Kir6.2 (Mouse, dilution 1/500)[2]. The expression of α-tubulin (dilution 1/20000; T5168, Sigma) was used as a loading control. After washing, the membrane was incubated for 1h in horseradish peroxidase coupled secondary antibodies (Goat Anti-Rabbit-HRP, Abcam, AB6721, dilution 1/8000) and the signal was detected using Amersham Hyperfilm ECL, after exposure to Amersham ECL WB Detection Reagent. Band intensity was measured using Image J [1].

Quantitative RT-PCR

RNA were isolated using RNeasy Mini kits with on-column DNase treatment (QIAGEN). cDNA were synthesized by High-Capacity cDNA Reverse Transcription Kit (Thermofisher). qPCR was conducted using TaqMan Gene Expression Assays (primer assay ID: ins1 Rn02121433_g1, Gcg Rn00562293_m1 and 18S 4310893E) in 7900 HT Fast Real-Time PCR System (Thermo Fisher Scientific). mRNA expression relative to housekeeping gene (18S) was determined by using the ΔΔCT method.

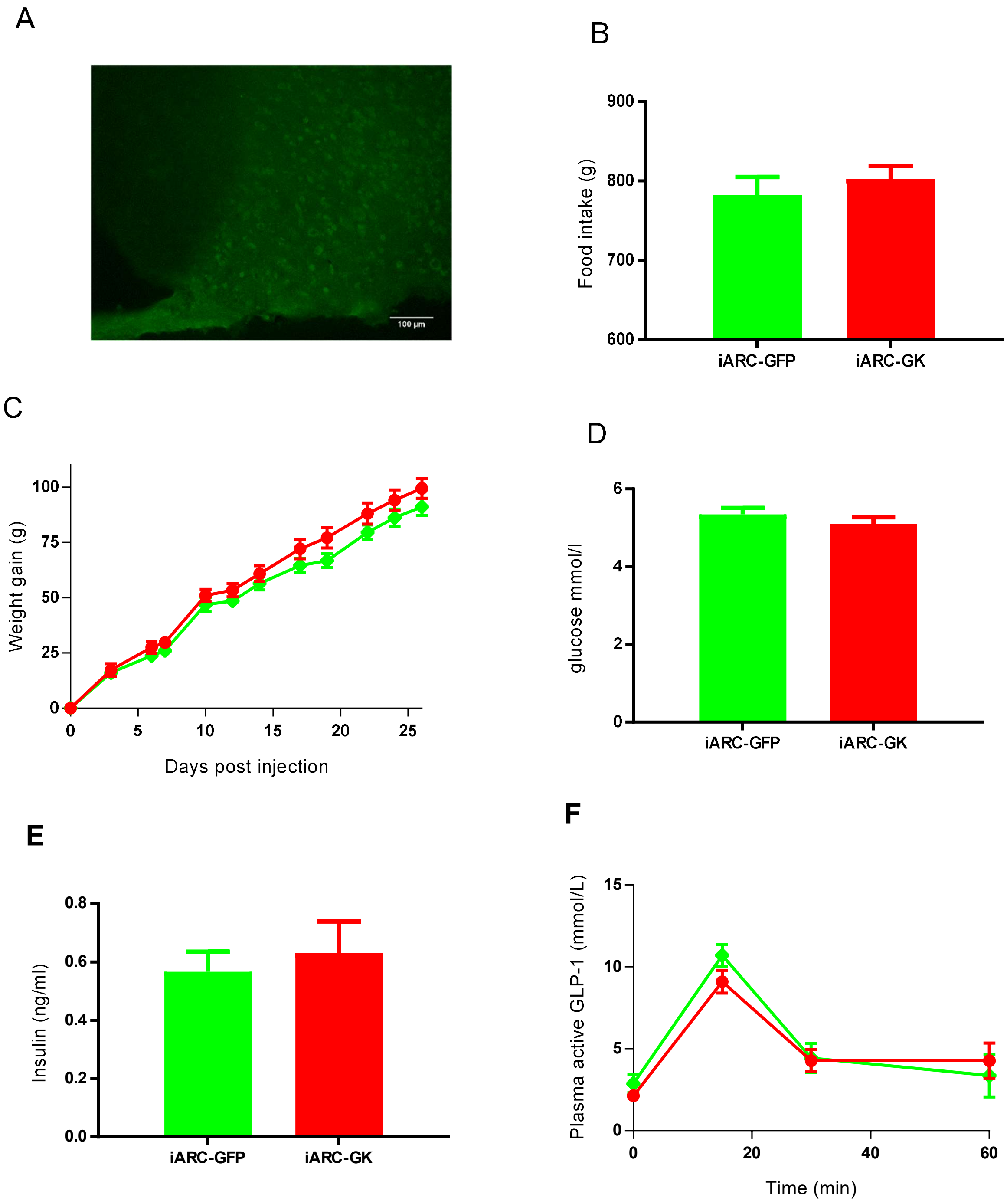

Supplementary Figure 1 Effect of chronically increased glucokinase activity in the arcuate nucleus
(A) Distribution of GFP detected by immunohistochemistry following intra-arcuate injection of rAAV-GFP
(B) Cumulative food intake in male Wistar rats on day 25 after intra-arcuate injection of either rAAV-GFP or rAAV-GK
(C) Body weight in male Wistar rats following intra-arcuate injection of either rAAV-GFP (green diamonds) or rAAV-GK (red circles)
(D) Fasting glucose following intra-arcuate injection of either rAAV-GFP (iARC-GFP) or rAAV-GK (iARC-GK)
(E) Fasting insulin following intra-arcuate injection of either rAAV-GFP (iARC-GFP) or rAAV-GK (iARC-GK)
(F) Active glp-1 during an oral glucose tolerance test in male Wistar rats following intra-arcuate injection of either rAAV-GFP (green diamonds) or rAAV-GK (red circles)
Data are mean ± SEM, n=10. Data for A and B were analysed by GEE, for C and D were analysed by t-test and for E was analysed by were analysed by ANOVA and post-hoc Holm-Sidak.

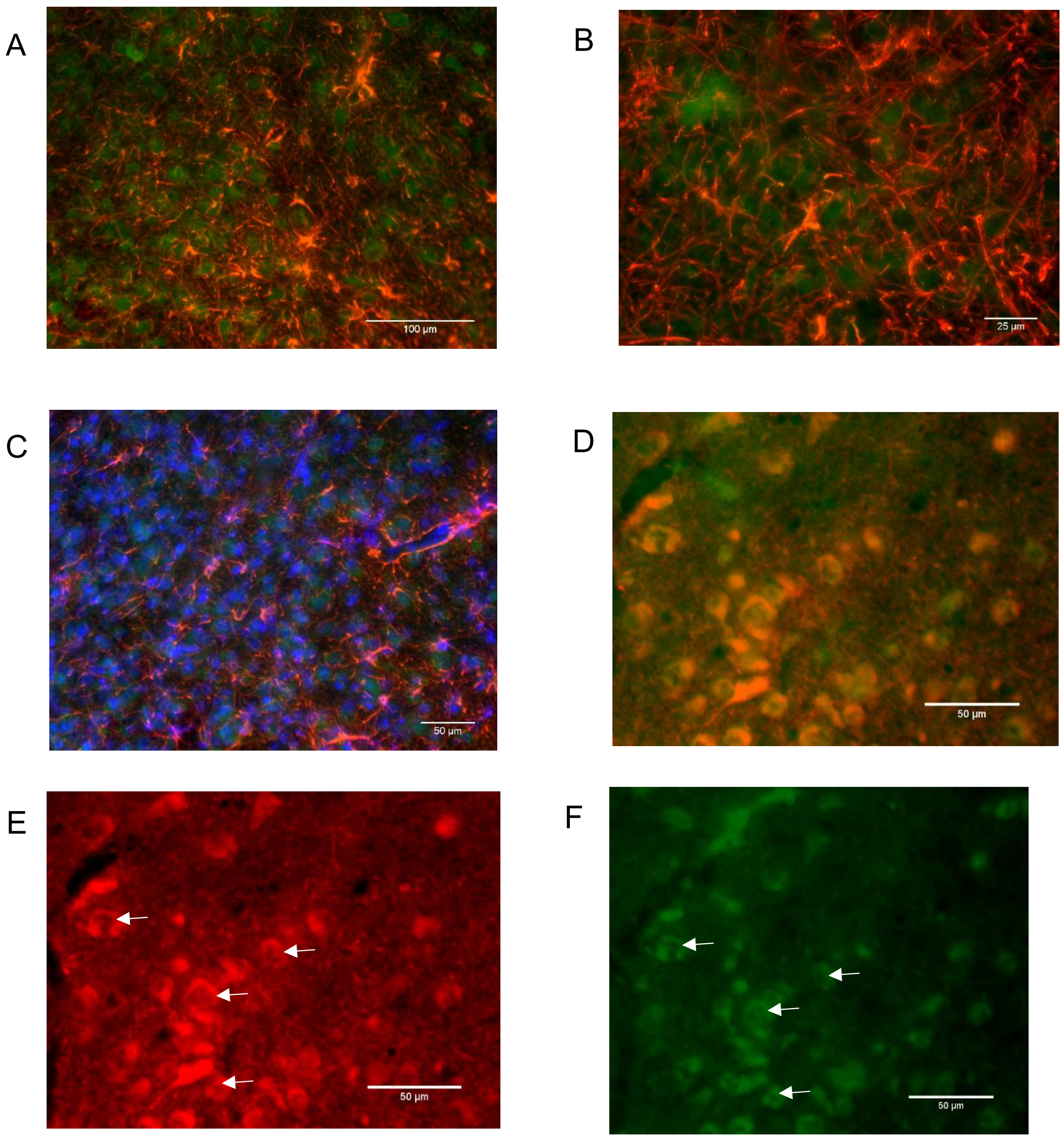

Supplementary Figure 2 Immunohistochemically localisation of GFP following rAAV injection into the arcuate nucleus.

(A) Immunohistochemical detection of both GFP (green) and GFAP (red) following injection of rAAV encoding GFP into the arcuate nucleus. There is clear separation of the GFP and GFAP with no overlap of expression of the two

(B) Immunohistochemical detection of both GFP (green) and GFAP (red) following injection of rAAV encoding GFP into the arcuate nucleus

(C)Immunohistochemical detection of both GFP (green) and GFAP (red) following injection of rAAV encoding GFP into the arcuate nucleus nuclei are counterstained with DAPI (blue)

(D) Immunohistochemical detection of both GFP (green) and neurone specific PGP9.5 (red) following injection of rAAV encoding GFP into the arcuate nucleus. Showing clear co-expression of the two proteins

(E) Immunohistochemical detection of neurone specific PGP9.5 following injection of rAAV encoding GFP into the arcuate nucleus. Arrows indicate some cells which also express GFP

(F) Immunohistochemical detection of GFP following injection of rAAV encoding GFP into the arcuate nucleus. Arrows indicate some cells which also express PGP9.5

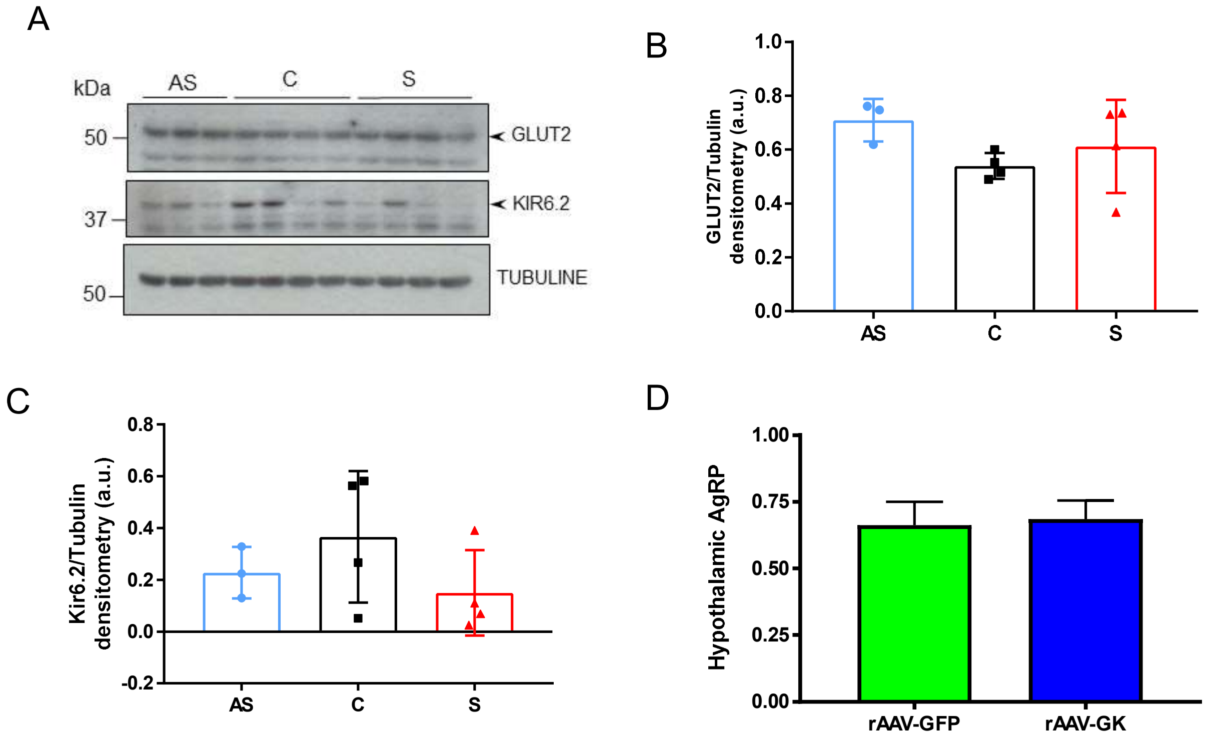

Supplementary Figure 3 Effect of genetically increased glucokinase activity in the arcuate nucleus on expression of other proteins.

(A) Western blot analysis of arcuate nucleus expression of GLUT2, KIR6.2 and tubulin as a loading control following intra-arcuate injection of either rAAV-GFP (C), rAAV-GK (S) or rAAV-AS-GK (AS)

(B) Quantification of western blot of arcuate nucleus GLUT2 expression corrected for loading using tubulin , following intra-arcuate injection of either either rAAV-GFP (C), rAAV-GK (S) or rAAV-AS-GK (AS)

(C) Quantification of western blot of arcuate nucleus Kir6.2 expression corrected for loading using tubulin , following intra-arcuate injection of either either rAAV-GFP (C), rAAV-GK (S) or rAAV-AS-GK (AS)

(D) Hypothlamic AgRP expression measured using qPCR, following intra-arcuate injection of either either rAAV-GFP (C), rAAV-GK (S) or rAAV-AS-GK (AS)

Data are represented as mean ± SEM, for A-C n=3 (antisense or 4 other groups). Data were analysed ANOVA with post Hoc Holm-Sidak. For D n=10 rAAV-GFP n=11 rAAV-GK, data were analysed using t-test.

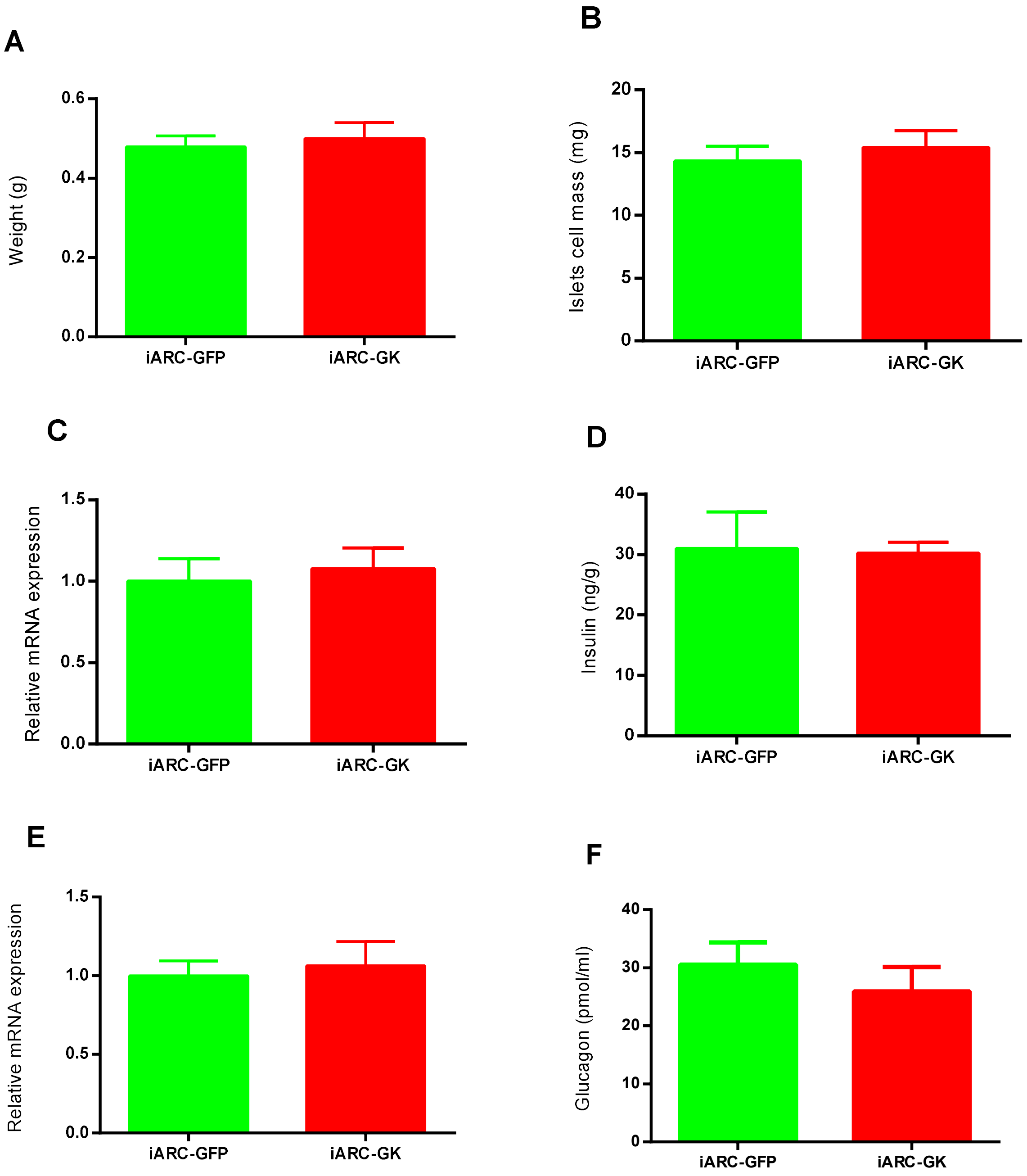

Supplementary Figure 4 Effect of genetically increased glucokinase activity in the arcuate nucleus on islet function.

(A) Pancreas weight following intra-arcuate injection of either rAAV-GFP (iARC-GFP) or rAAV-GK (iARC-GK)
(B) Islet mass, following intra-arcuate injection of either rAAV-GFP (iARC-GFP) or rAAV-GK (iARC-GK)
(C) Pancreatic insulin expression following intra-arcuate injection of either rAAV-GFP (iARC-GFP) or rAAV-GK (iARC-GK)
(D) Pancreatic insulin content following intra-arcuate injection of either rAAV-GFP (iARC-GFP) or rAAV-GK (iARC-GK)
(E) Pancreatic glucagon expression following intra-arcuate injection of either rAAV-GFP (iARC-GFP) or rAAV-GK (iARC-GK)
(F) Pancreatic glucagon content following intra-arcuate injection of either rAAV-GFP (iARC-GFP) or rAAV-GK (iARC-GK)
Data are represented as mean ± SEM, n=10. Data were analysed by t-test.

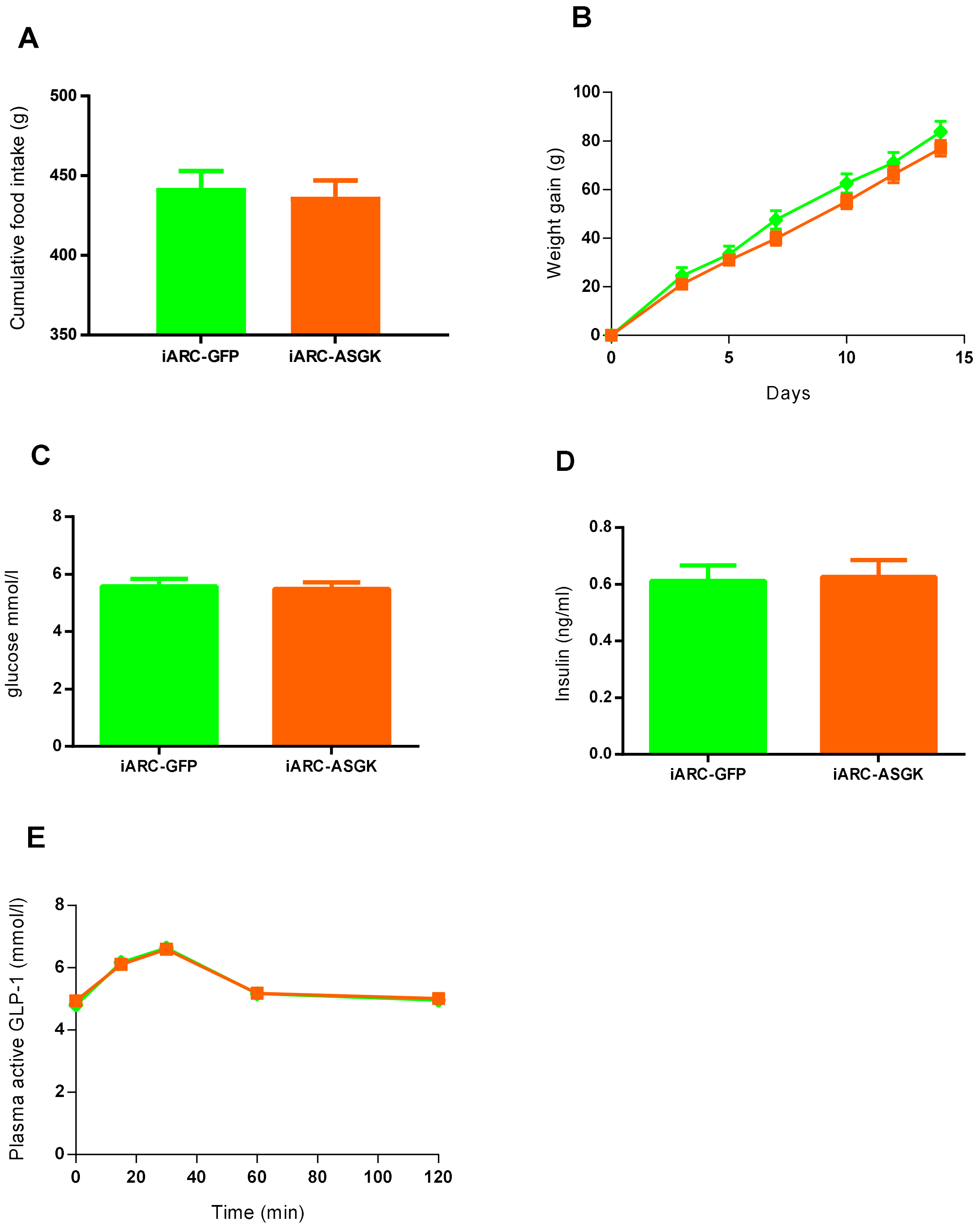

Supplementary Figure 5 Effect of chronically decreased glucokinase activity in the arcuate nucleus
(A) Cumulative food intake in male Wistar rats 15 days following intra-arcuate injection of either rAAV-GFP or rAAV-ASGK
(B) Body weight in male Wistar rats following intra-arcuate injection of either rAAV-GFP (green diamonds) or rAAV-ASGK (orange squares)
(C) Fasting glucose following intra-arcuate injection of either rAAV-GFP (iARC-GFP) or rAAV-GK (iARC-ASGK)
(D) Fasting insulin following intra-arcuate injection of either rAAV-GFP (iARC-GFP) or rAAV-GK (iARC-ASGK)
(E) Active glp-1 during an oral glucose tolerance test in male Wistar rats following intra-arcuate injection of either rAAV-GFP (green diamonds) or rAAV-ASGK (orange squares)
Data are mean ± SEM, n=10. Data for A and B were analysed by GEE, for C and D were analysed by t-test and for E was analysed by were analysed by ANOVA and post-hoc Holm-Sidak.

References

1 Schindelin J, Arganda-Carreras I, Frise E*, et al.* Fiji: an open-source platform for biological-image analysis. Nat Methods. 2012; **9**: 676-682

2 Arakel EC, Brandenburg S, Uchida K*, et al.* Tuning the electrical properties of the heart by differential trafficking of KATP ion channel complexes. J Cell Sci. 2014; **127**: 2106-2119
